# Phylogenetic conservation in plant phenological traits varies between temperate and subtropical climates in China

**DOI:** 10.3389/fpls.2024.1367152

**Published:** 2024-04-10

**Authors:** Khurram Shahzad, Mengyao Zhu, Lijuan Cao, Yulong Hao, Yu Zhou, Wei Liu, Junhu Dai

**Affiliations:** ^1^ Key Laboratory of Land Surface Pattern and Simulation, Institute of Geographic Sciences and Natural Resources Research, Chinese Acadamy of Sciences (CAS), Beijing, China; ^2^ University of Chinese Academy of Sciences, Beijing, China

**Keywords:** geographical regions, plant phenology, plant functional traits, phylogenetic conservation, temperate and subtropical climates

## Abstract

Phenological traits, such as leaf and flowering dates, are proven to be phylogenetically conserved. The relationship between phylogenetic conservation, plant phenology, and climatic factors remains unknown. Here, we assessed phenological features among flowering plants as evidence for phylogenetic conservatism, the tendency for closely related species to share similar ecological and biological attributes. We use spring phenological traits data from 1968-2018 of 65 trees and 49 shrubs in Xi’an (temperate climate) and Guiyang (subtropical climate) to understand plant phenological traits’ relationship with phylogeny. Molecular datasets are employed in evolutionary models to test the phylogenetic conservatism in spring phenological characteristics in response to climate-sensitive phenological features. Significant phylogenetic conservation was found in the Xi’an plant’s phenological traits, while there was a non-significant conservation in the Guiyang plant species. Phylogenetic generalized least squares (PGLS) models correlate with phenological features significantly in Xi’an while non-significantly in Guiyang. Based on the findings of molecular dating, it was suggested that the Guiyang species split off from their relatives around 46.0 mya during the middle Eocene of the Tertiary Cenozoic Era, while Xi’an species showed a long evolutionary history and diverged from their relatives around 95 mya during the late Cretaceous Mesozoic Era. First leaf dates (FLD) indicative of spring phenology, show that Xi’an adjourned the case later than Guiyang. Unlike FLD, first flower dates (FFD) yield different results as Guiyang flowers appear later than Xi’an’s. Our research revealed that various factors, including phylogeny, growth form, and functional features, influenced the diversity of flowering phenology within species in conjunction with local climate circumstances. These results are conducive to understanding evolutionary conservation mechanisms in plant phenology concerning evolutionary processes in different geographical and climate zones.

## 1 Introduction

Phylogenetic conservatism (closely related species tend to show similar traits) might be the biological basis for specific phenological events in plants or sensitivity to abiotic environmental factors (Davies et al., 2013; CaraDonna et al., 2014; Yang et al., 2021). Still, it is poorly understood how evolutionary mechanisms and geological and climatic conditions influence plant phenology and phylogeny relationships. Numerous studies focus more on the interannual variation of phenology that is affected by climatic factors (such as temperature, precipitation, and daylight) (Ge et al., 2015; Piao et al., 2019). In contrast, many studies demonstrated that plants are now leafing out earlier and flowering earlier in response to a warming climate (Wolkovich et al., 2012; Bucher et al., 2018; Menzel et al., 2020; Rosbakh et al., 2021). Climate change is due to geological changes such as latitudinal range (Hickling et al., 2006; Mason et al., 2015) and elevational extent (Chen et al., 2011). However, recent research suggested that the biological foundation for phenological occurrences in some plants or sensitivity to abiotic environmental stimuli may be related to plant phylogeny, which states that closely related species tend to exhibit comparable phenological properties (Caradonna and Inouye, 2015; Yang et al., 2021). This might undermine evolutionary conservatism by causing variations in phenological timing or temperature sensitivity among closely related species. Phylogenetic conservation may be obscure because the same biological groups may encounter various environmental limitations and have followed different evolutionary pathways (Davies et al., 2013; Du et al., 2015). Therefore, it is essential to accurately improve the future forecast of how geographical and environmental factors combined with phylogenetic conservatism impact plant phenophases.

Changes in plant phenology are not uniform across the globe due to the variance in plant phenological sensitivity to climate change (Menzel et al., 2006; Gao et al., 2019). Spatial variance in plant phenology change rate can modify well-established phenological patterns along geographical gradients (Ma et al., 2018; Vandvik et al., 2018; Liu et al., 2019). There are substantial spatial variations because of regional geographical conditions affecting climate (van der Wiel and Bintanja, 2021). For example, surprising regional differences with local hotspots have been identified in temperature changes across the United States (Eilperin et al., 2020). The constraint of phylogenetic conservation change in plant phenological features due to geographical and climatic zones must be understood at a regional scale (i.e., climate differences and geographical variables) (Alice Boyle and Bronstein, 2012). Relatively little attention has been paid to the potential impacts of geographical variables (such as latitude and altitude) that influence climatic variability, which will have consequences in phenological and phylogenetic conservation. By studying these variables at different spatial and temporal scales, the effects of climate and geography on the evolutionary mechanisms influencing phylogenetic conservatism in plant phenophases will be more correctly predicted.

Phylogenetic conservation contributes to plants by clarifying taxonomic status, identifying unique evolutionary lineages, determining relictual and recently derived species, and investigating the phylogenetic value for conservation priority between regional and widespread species (Coates, 2000; Ryder, 1986). The phylogenetic conservatism in plant phenophases in response to climatic sensitivity has been well-reported (Davies et al., 2013; Caradonna and Inouye, 2015; Du et al., 2015). However, the mechanism behind how climatic factors would add considerable uncertainty and affect the relationship between plant phenology and phylogeny is still unknown. For a meaningful prediction of plant phylogeny and phenology correlations with regional climatic differences, an ability to consider attributes of shared evolutionary history is essential (Cleland et al., 2012; Wolkovich and Cleland, 2014).

The phylogenetic conservatism of phenological features has since been evaluated using various techniques, such as Blomberg’s K and Pagel’s lambda methods (Blomberg et al., 2003; Münkemüller et al., 2012; Davies et al., 2013; Li et al., 2016). Significant efforts were made to gather phenological records and plant characteristic data from national flora books to know the relationship between plant phenology and phylogeny. However, the accuracy of these studies’ phenological and genetic data is not very high (Du et al., 2015, 2017). The extent of these studies ranged from three years (Basnett et al., 2019) to a few decades (Davies et al., 2013; Du et al., 2017; Yang et al., 2021), which varied greatly in these studies. Using incomplete genetic and phenological datasets can cause incongruence in the phylogenetic signals in plant phenophases. The comparison of chloroplast genome (cpDNA) sequences among different plant species is an essential source of plant molecular phylogenetic data, making it an ideal molecule for tracing the evolutionary history of plant species (Chen et al., 2022). Here, we use the complete chloroplast genome (cpDNA) dataset to reconstruct the phylogenetic gene genealogies of plant species to better understand the phylogenetic conservation between plant phenological and climatic sensitive phenological traits.

Using plant functional traits (the characteristics of plants that determine responses in the surrounding environment, other species, and trophic levels) has become an efficient and accurate way to investigate the effects of large-scale land and climate change (Díaz and Cabido, 2001; Suding et al., 2008; Pérez-Harguindeguy et al., 2016). The functional traits of plants are essential biological characteristics that most likely reflect the adaptation strategies of plants to the environment (Funk et al., 2017). However, our understanding of the confounding influences of plant functional traits (i.e., life forms, pollination style, deciduous, and evergreen) affect plant phenology and phylogenetic conservation remains unclear. There is evidence that differences in plant functional traits, such as life form, and biotic and abiotic pollination mode, may also be associated with interspecific variation in plant phenology (Wolkovich and Cleland, 2014; Du et al., 2017; Bucher et al., 2018; Liu et al., 2021). However, some studies also found there were no significant differences in the flowering time of the entomophilous plants (Janeček et al., 2021). Therefore, the relationship between functional traits and plant phenology still deserves further exploration. This study analyzed the relationship between plant functional traits and the spring phenological characteristics of species in two different geographical regions.

Climate determines the reproductive phenology of plants, such as temperature and precipitation cause various reproductive structures to grow and mature (Rathcke and Lacey, 1985; Høye et al., 2007; Wang et al., 2023). Ting et al. (2008) concluded that the fruiting period is progressively shorter with increasing latitude because the climate varies with the geographical gradient. On the other hand, our analysis may become highly ambiguous if we ignore the long-term variations in geography and climate that affect the length of reproductive phenology. Recent studies on the phenology of common alder are consistent with the idea that local geographical climate variation affects phenology (Ziello et al., 2009; Wang et al., 2023). For instance, China’s average annual rainfall gradually drops as northern latitude rises and east longitude falls (Xu and Zhang, 2020), shortening the time that photosynthesis (Huxman et al., 2004). Another illustration is when a species cannot break the dormant state of its seeds due to an environment that is too warm for it. On the other hand, frost damage can occur to plants that flower early or prematurely (Morin et al., 2007). Thus, the length of reproductive phenology may be shortened. Consequently, a thorough account of the global distribution and spatial patterns of the duration of reproductive phenology is needed. Therefore, it’s equally essential to comprehend how plants might react to climate change.

This study investigates the phylogenetic conservatism in spring phenological characteristics and the functional traits correlations with phenological elements such as life forms (trees and shrubs) and evergreen or deciduous species in two different climatic conditions zones, i.e., Xi’an (temperate climatic) and Guiyang (subtropical climatic) in China. The phylogenetic signal and evolutionary models analyzed phylogenetic conservation in plant phenological traits. It should be noted that because a small sample size may reduce the predicted accuracy of the phenological model, we excluded species with less than 50 years of flowering and leaf-out data. To accomplish our objective, we address the following questions: (i) To explore the phylogenetic conservation between plant spring phenophases and climatic-sensitive spring phenophases of two different climatic zones, i.e., Xi’an and Guiyang in China. (ii) How do temperate and subtropical climatic conditions influence the phylogenetic signals in plant phenological traits of Xi’an and Guiyang species? (iii) To investigate different geographical and climatic conditions that are directly connected to the area’s evolutionary processes, which strongly impact plant spring phenology and phylogeny relationships.

## 2 Materials and methods

### 2.1 Study sites

**Figure 1:**
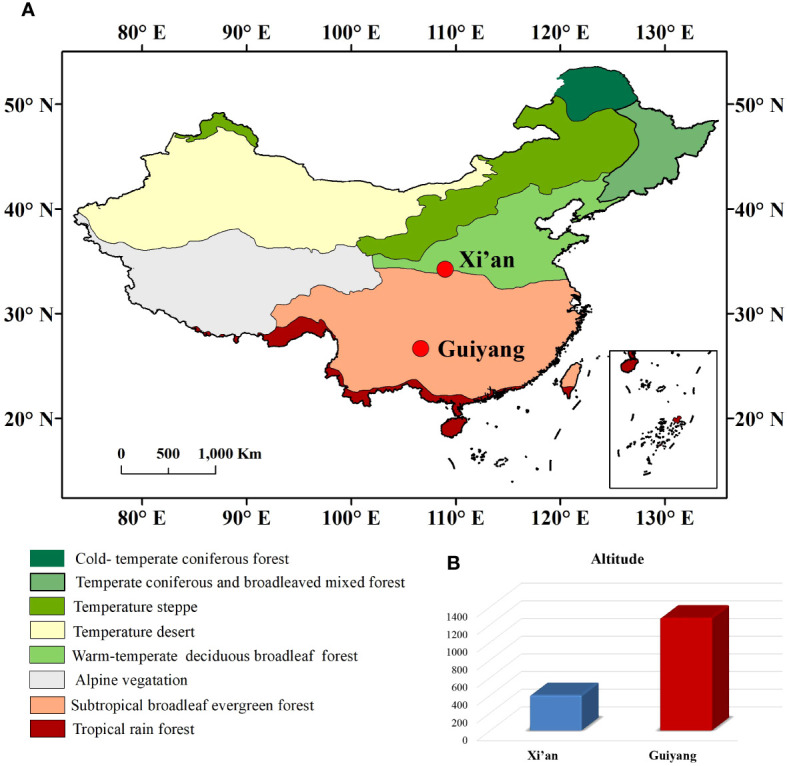
**(A)** Representation of geographical map with all climatic zones of China and the location of Xi’an and Guiyang. **(B)** The altitude difference between Xi’an and Guiyang.

**Figure 2:**
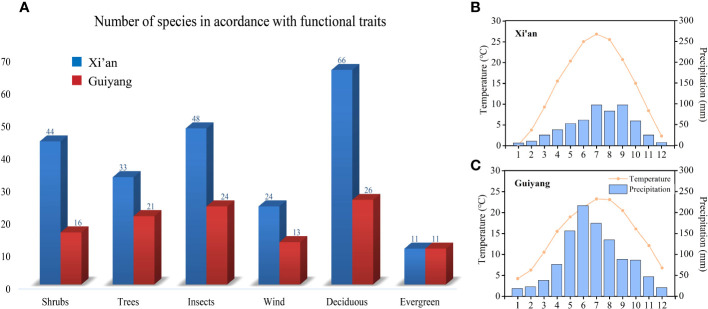
**(A)** Graphical overview of shrubs and trees; wind and insect-pollinated species; deciduous and evergreen species in Xi’an and Guiyang **(B)** Temperature and precipitation graph of Xi’an **(C)** Temperature and precipitation graph of Guiyang.

Xi’an and Guiyang are two historical regions of China with different geographical and climatic conditions. Xi’an (34°12’N, 108°57’E) is the capital of the Shaanxi province located in north-central China (Bai et al., 2010). Xi’an (400m a.s.l) has a temperate semi-humid climate with an average temperature of 13°C and average precipitation of 578 mm annually (Bai et al., 2010). The vegetation in Xi’an is sharply differentiated into northern and southern zones with mixed deciduous broad-leaved and evergreen forests. Guiyang (26°38’N and 106°37’E) is the city of Guizhou Province, located in southwestern China. Guiyang is a humid subtropical climatic region at 1,050-1,275m a.s.l (**Figure 1**). Due to its high altitude, the annual temperature is 15.3°C, and rainfall is about 11,000mm (**Figure 2**). Guiyang City typically has harsh climatic conditions (subtropical climate), such as high relative humidity, long, cloudy, rainy days, and little sunshine. Natural wealth lies in its forests; about one-tenth of the land is under natural forest. It has rich and valuable woodlands of wild plants, among which several highly valued herbs are used in traditional Chinese medicine.

### 2.2 Phenological and meteorological data observation

The phenological observation records of the first leaf dates (FLD) and first flower dates (FFD) of 77 and 37 plant species from Xi’an and Guiyang respectively have been collected from the China Phenological Observation Network (CPON). The details of plant species and functional traits (life forms and evergreen or deciduous species) are given in **Supplementary Table 1**. The phenological observation data at each site (Xi’an and Guiyang) were collected from 1968-2018, followed by defined observation criteria and procedures (Wan and Liu, 1979). According to observational standards, the first leaf out and flowering dates are determined as a fixed individual plant of a specific species starts generating the first leaf and the first flower, respectively (Dai et al., 2013; Wang et al., 2015). We then compared differences in phenophases (FLD and FFD) and their deviations in the period 1968-2018 between Xi’an and Guiyang species.

The meteorological data is extracted from the China Meteorological Data Service Center website (https://data.cma.cn/) to acquire daily mean temperatures and precipitation from 1963 to 2018 for each station. We used the daily mean temperatures and precipitation data to calculate spring phenology’s temperature and precipitation sensitivity.

### 2.3 Molecular phylogenetics and divergence time analysis

The available complete chloroplast genomes (cpDNA) of selected Xi’an and Guiyang station plants were downloaded from GenBank (NCBI accession numbers and species details presented in **Supplementary Table 1**). *Agaricus bisporus* and *Cantharellus cibarius* complete genomes are added as outgroups. Before phylogenetic analysis, each station dataset is aligned separately using Genious v 12.0 software and the multiple alignment application MAFFT (Darling et al., 2004). We directly generated an alternate phylogeny from DNA sequence data to test the sensitivity of our findings to the tree topology. Further details of tree reconstruction are provided as supplementary information (**Supplementary Information Index II**). We refer to the final topology as the ML tree.

In BEAST v1.8.0, the multiple fossil calibrations (**Supplementary Information Index I**) were used to estimate the divergence periods between lineages with a relaxed clock and Yule process speciation prior (Near et al., 2005; Marshall, 2008; Drummond et al., 2012; Lukoschek et al., 2012). MrModeltest 2.3 was used to choose the GTRAGMMA nucleotide substitution model (Nylander, 2004). In this case, we considered the uncertainty of prior knowledge using a normal prior probability distribution. The parameters were sampled every 5,000 generations, while the analysis was run for 20,000,000 generations. Using Tracer v. 1.6 (Drummond et al., 2012), the appropriate sample size (>200) was established, and the first 10% of the samples were eliminated as burn-ins. To construct a maximum clade credibility chronogram depicting the mean divergence time estimates with 95% highest posterior density (HPD) intervals, we used Tree Annotator v.1.8.0 (Drummond et al., 2012) to compile the collection of post-burn-in trees and associated parameters. FigTree V1.3.1 (Drummond et al., 2012) displayed the resulting divergence times.

### 2.4 Phylogenetic conservatism in spring phenology

For phylogenetic conservatism, phylogenetic topology was the resort. The following analyses were carried out using the “ape” (Paradis et al., 2004) and “picante” (Kembel et al., 2010) libraries in R (http://www.R-project.org; R Development Core Team). Blomberg’s K technique was used to analyze each station’s phylogenetic signal using spring phenological variables (Blomberg et al., 2003; Gao et al., 2022). A trait’s evolution is influenced by phylogeny if K=1 shows that the inter-species correlation equals the Brownian expectation. According to Brownian motion (BM), K>1 demonstrates that trait similarity is more significant than expected (Blomberg et al., 2003). In contrast, K<1 denotes either stability (i.e., the characteristic is phylogenetically conserved) or absence of phylogenetic structure (i.e., the trait is not phylogenetically conserved) (Wiens et al., 2010). Using the *phytools* library in the R, we calculated the K parameter for each phenological characteristic. To determine whether the observed values significantly deviate from the randomized arrangement. The P-value might alternatively be derived by 1000 interactions in the computation of K (Revell, 2012).

We approached the close fitting of evolutionary models for evaluating phylogenetic conservatism as a random variation and evolutionary stasis shaped by selection can be directly captured by the white noise (WN) model and Ornstein-Uhlenbeck (OU) model, respectively (Felsenstein, 1985; Blomberg et al., 2003; Butler and King, 2004; Kozak and Wiens, 2010; Diniz-Filho et al., 2015). Phylogenetic signal representation (PSR) curves to investigate the evolutionary patterns of trait development (Diniz-Filho et al., 2012). We assessed each trait’s evolutionary processes by comparing the close fitting of the three most popular evolutionary models (BM, OU, and WN) with the highest value of weight Akaike Information Criterion model to make up for the limitations of the phylogenetic signal approach (wAIC) (Butler and King, 2004; Diniz-Filho et al., 2012). Phylogenetically independent trait variation was modeled using the WN model with random variation, the BM model of progressive drift, and the OU model for stasis or stable selection. It is predicated on the phylogenetic eigenvector regression (PVR) model, which employs eigenvectors obtained and chosen from a pairwise phylogenetic distance matrix to describe trait variation. To construct PSR curves, we followed Staggemeier et al. (2015) methods to know the evolution and conservation of phenological traits. Additionally, the PSR curve’s shape reflects the pace of trait evolution in the phylogenetic tree (Diniz-Filho et al., 2012).

### 2.5 Sensitivity of plant phenology and phylogenetic conservation

The spring phenological traits sensitivity analysis to temperature and precipitation was performed differently between Guiyang and Xi’an stations. To identify the preseason, we determined the Pearson’s correlation coefficient (r) between FLD/FFD and temperature during the 1 day, 2 days,…, and 120 days before the average FLD/FFD of the study period, respectively. The preseason was then identified as the period with the highest “r” (Dai et al., 2019). The regression slope between the FLD/FFD and the daily mean temperature averaged throughout the preseason determined the temperature and precipitation sensitivity. The above method (section 2.4) was applied to know the phylogenetic conservation in the sensitivity of plant phenology.

We performed the frequency distribution analysis to compare the temperature sensitivity as days and precipitation sensitivity as days/mm to check the advancement of the Xi’an and Guiyang species in spring phenology (FLD and FFD).

### 2.6 Statistical analysis

This paper mainly analyzes the influence of three functional traits, i.e., life form (shrubs and trees), pollination style (biotic and abiotic), and distinct plant groups (deciduous and evergreen), on spring phenological traits (**Figure 2**). Refer to “Flora of China” for functional traits classification: life form; trees whose maximum height exceeds six meters are classified as trees, and those whose size does not exceed six meters are grouped as shrubs. Pollination methods: gymnosperms and angiosperms with small, odorless flowers and many stamens are classified as anemophilous plants; angiosperms with large, fragrant flowers, conspicuous petals, and brightly colored flowers are grouped as insect-borne plants (Du et al., 2017). Deciduous plants are considered a group of plants that shed their leaves seasonally, while evergreen plants are considered a group of plants that keep their leaves throughout the entire year. We used phylogenetic generalized least squares (PGLS) models to compensate for phylogenetic autocorrelation. The ‘*pgls*’ function from the Caper R package fits *PGLS* models (Orme et al., 2014). This allowed us to study the impacts of various plant function features on spring phenological traits in temperature and precipitation variations. **Figure 2** represents the functional traits and numbers of species in Guiyang and Xi’an. We performed the mean and median range analysis to compare the plant functional features such as tree and shrub species, biotic and abiotic pollination, and deciduous and evergreen species between the spring phenological traits (FLD and FFD, temperature and precipitation sensitivities of FLD and FFD) of Guiyang and Xi’an.

## 3 Results

### 3.1 Plant phenological characteristics in Guiyang and Xi’an areas

**Figure 3:**
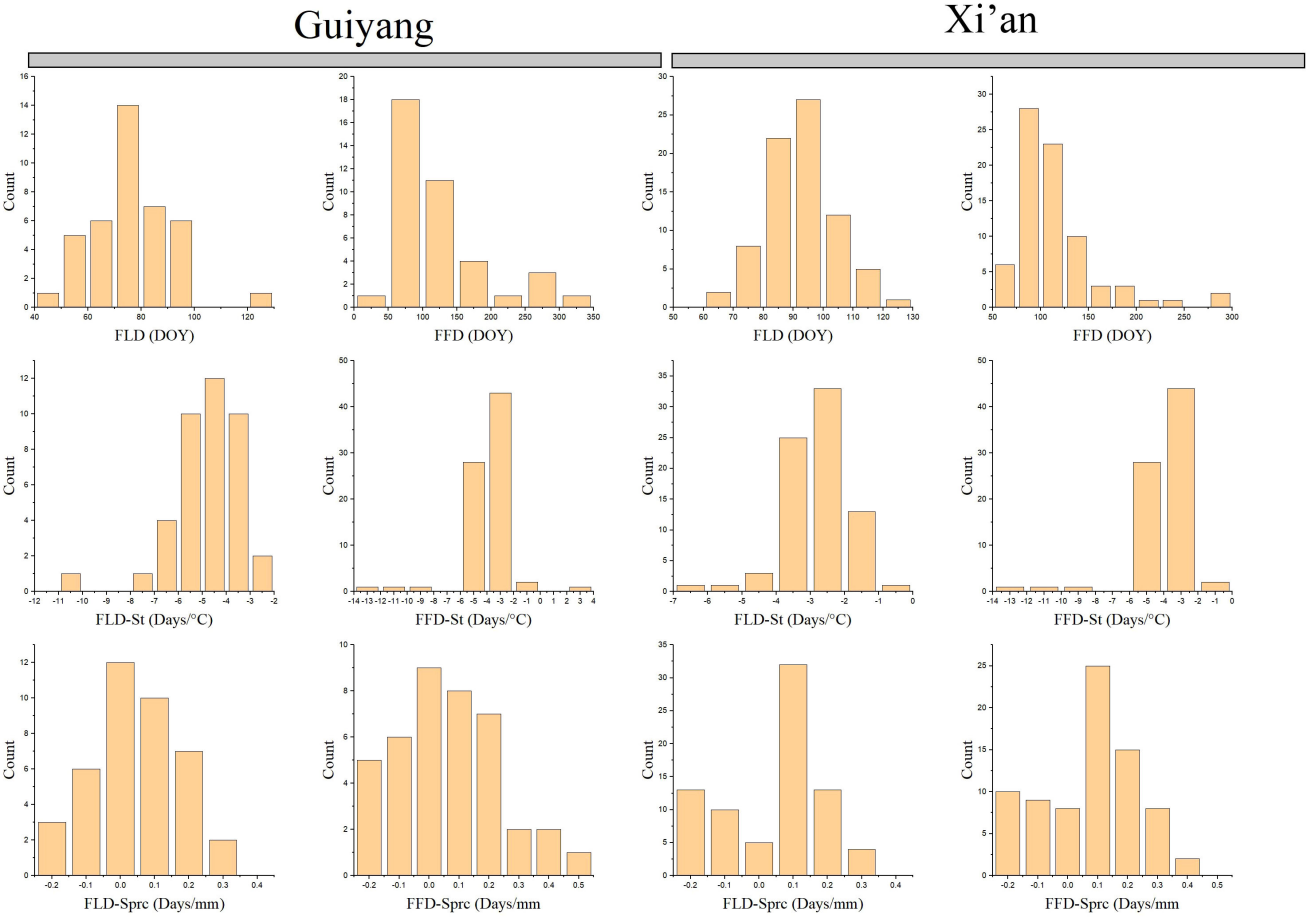
Spring phenophases of plant species collected from 1968 to 2018 in Guiyang and Xi’an. FLD, first leaf date; FFD, first flower date; FLD-S_T_, first leaf date temperature sensitivity; FFD-S_T_, first flower date temperature sensitivity; FLD-S_Prc_, first leaf date precipitation sensitivity; FFD-S_Prc_, first flower date precipitation sensitivity, respectively. DOY, Day of the year.

Our results compare the spring phenological characteristics and the differences between each Guiyang and Xi’an species. Guiyang’s first leaf dates (FLD) indicate that leafing begins at 40 days and lasts until 100, while Xi’an FLD findings suggest that later leafing starts after 60 days and lasts until 120 (**Figure 3**). Overall, Xi’an adjourned the case later than Guiyang. The first flower dates (FFD) produce distinct outcomes compared to FLD. While Xi’an’s FFD results show later leaves beginning before 50 days and ending at 250 days, Guiyang’s results show blooming flowers starting at 50 days and lasting more than 300 days. Typically, Guiyang flowers are later than that of Xi’an.

The spring phenological traits sensitive to temperature and precipitation reveal that Guiyang’s temperature sensitivity leaf phenology (FLD-S_T_) begins leafing when the temperature is between -8 and -2 °C. However, according to FLD-S_T_ data, plants from Xi’an begin to leaf at temperatures between -5 and -1 °C (**Figure 3**). In general, Xi’an has a lower leaf-out temperature than Guiyang. On the other hand, species from Guiyang and Xi’an commence flowering with an average temperature of -14 to -2 °C according to the temperature sensitivity of the first flower date (FLD-S_T_) (**Figure 3**). In Guiyang and Xi’an species, the first leaf date (FLD-S_prc_) precipitation sensitivity exhibits the same pattern, with an average precipitation of -0.2 to 0.3mm before leafing begins. In contrast, first flower date (FFD-S_prc_) precipitation sensitivity analysis indicated that average rainfall starts flowering at -0.2 to 0.5mm in Guiyang. The FFD-S_prc_ findings for the Xi’an species show that flowering begins at -0.2 to 0.4mm in precipitation. Overall, Xi’an experiences less blooming than Guiyang.

### 3.2 Phylogenetic conservation in plant phenology and sensitivity of plant phenology

The K-value was below one for all the spring phenological characteristics in Xi’an and Guiyang, predicted by Brownian motion. However, the signal intensity was different between the two locations. The leaf and flower phenological characteristics show weaker and non-significant phylogenetic signals (K values) in Guiyang. Therefore, the phylogenetic conservation signals in the spring phenological characteristics (FLD, FLD-S_T_, FLD-S_prc_, FFD, FFD-S*T*, FFD-S_prc_) of Xi’an species were solid and significant (**Table 1**). The WN model had the lowest wAIC value among the three evolutionary models, demonstrating that all the spring phenological traits were not preserved phylogenetically in Guiyang (**Table 1**). The OU model’s lower wAIC number for all Xi’an phenological traits indicates that the evolution of spring phenological qualities was slower than predicted by the Brownian model. However, the findings from three evolutionary models of Xi’an show a similar pattern with substantial conservation, demonstrating that the degree of phylogenetic conservatism in phenological traits has recently changed.

**Table 1:** Phylogenetic conservation signals in spring phenophases and response to climatic sensitivity (temperature and precipitation).

No.	Name of Phenophases	Blomberg’s K value	P Value	BM	OU	WN
Guiyang						
1	FLD	0.13	0.367	370.7142	335.716	333.3738*
2	FFD	0.12	0.463	478.10	462.64	461.87*
3	FLD-S_T_	0.11	0.547	186.47	151.43	149.15*
4	FFD-S_T_	0.09	0.646	241.212	218.061	215.7854*
5	FLD-S_Prc_	0.08	0.83	291.75	278.93	274.89*
6	FFD-S_Prc_	0.10	0.66	321.42	277.96	275.34*
Xi’an						
1	FLD	0.228	0.165	615.6328	598.8631*	603.7633
2	FFD	0.209	0.165	829.7437	807.9478*	808.2966
3	FLDS_T_	0.207	0.217	232.9588	210.1808*	213.7730
4	FFDS_T_	0.195	0.169	405.8885	382.4478*	383.6406
5	FLD-S_Prc_	0.09	0.641	214.503	191.2332*	265.2623
6	FFD-S_Prc_	0.133	0.128	224.303	199.1742*	214.5212

FLD, first leaf date; FFD, first flower date; FLD-S_T_, first leaf date temperature sensitivity; FFD-S_T_, first flower date temperature sensitivity; FLD-S_Prc_, first leaf date precipitation sensitivity; FFD-S_Prc_, first flower date precipitation sensitivity; WN, white noise model; OU, Ornstein-Uhlenbeck model; and BM, Brownian motion. * Means significant conservative value.

### 3.3 Phylogenetic signals of each species (tree topology) in plant spring phenophases

**Figure 4:**
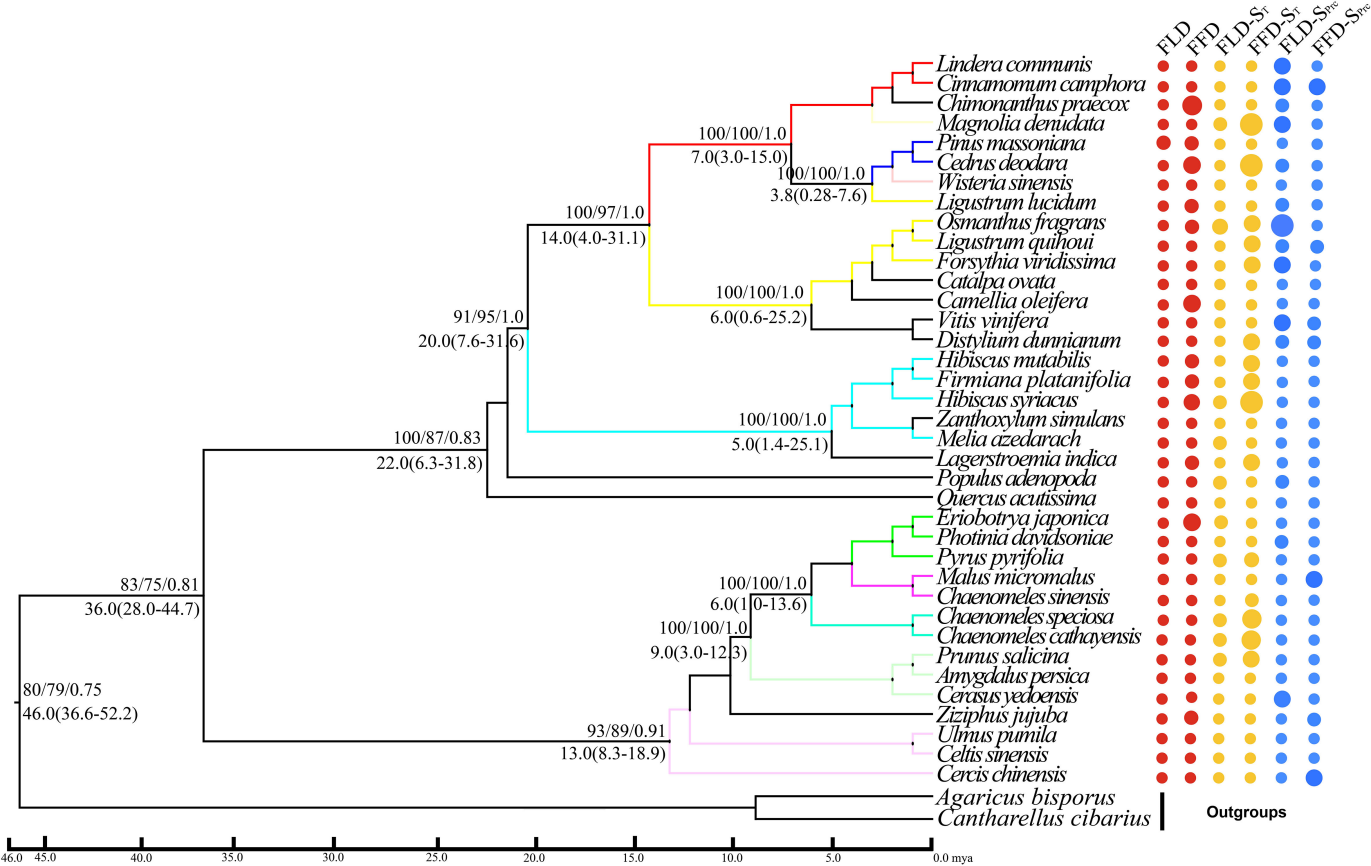
Phylogenetic signals of phenological traits in Guiyang station. A plot of spring phenological attributes at the tips of the phylogenetic tree of 37 species in Guiyang. FLD; first leaf date, FFD; first flower date, FLD-ST; first leaf date temperature sensitivity, FFD-ST; first flower date temperature sensitivity, FLD-SPrc; first leaf date precipitation sensitivity, FFD-SPrc; first flower date precipitation sensitivity, respectively. The sizes of the circles are proportional to the values of the traits, i.e., a larger size indicates later FLD or FFD, a larger size indicates stronger temperature sensitivity, and a larger size means FLD-SPrc is more sensitive with precipitation. Bootstrap values of Neighbor Joining, Maximum Likelihood and Bayesian Interference are written above each branch respectively. Molecular divergence time and 95% HPD values are written below each branch.

**Figure 5:**
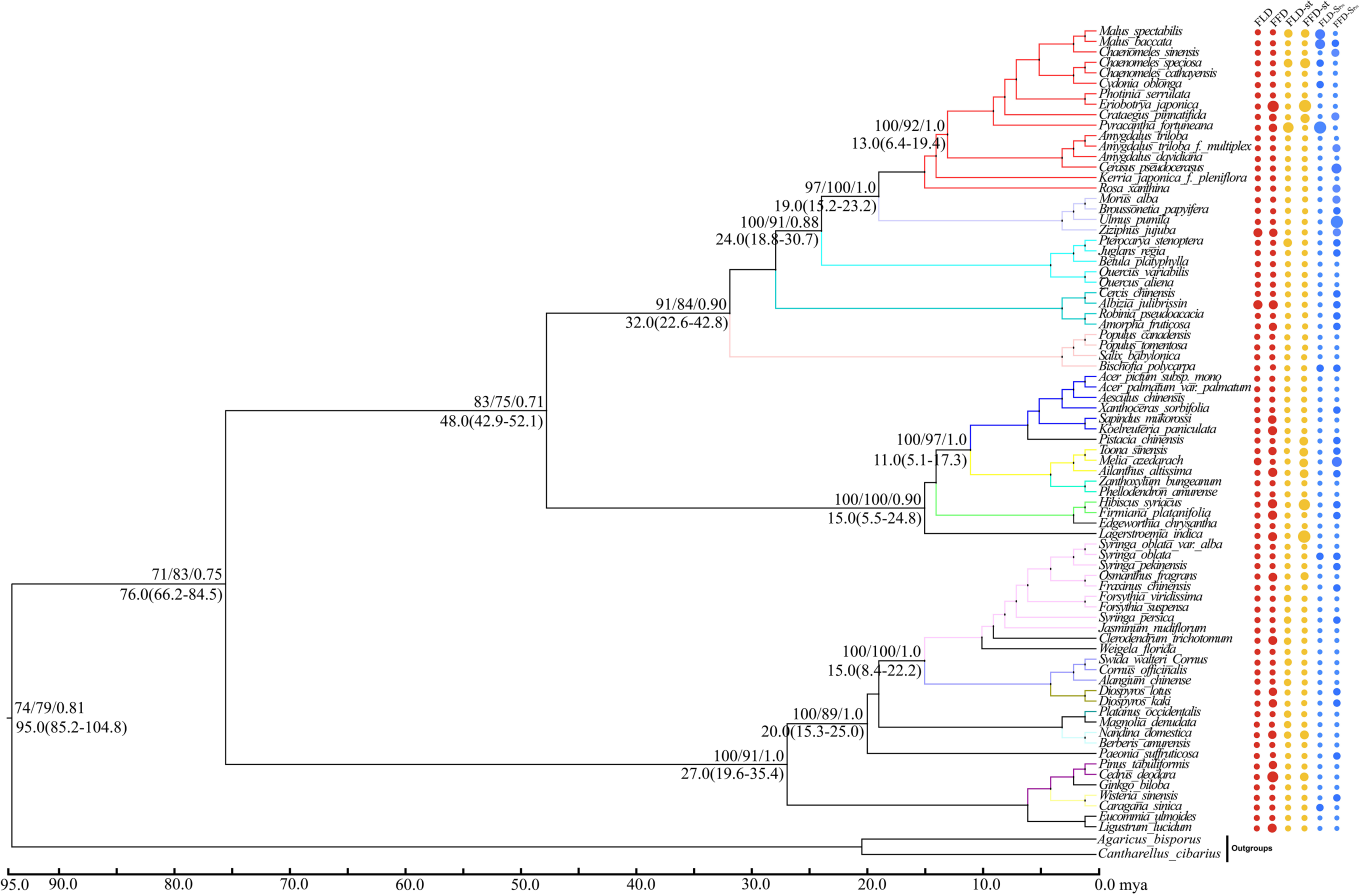
Topology diagram and phylogenetic signals of phenological traits in Xi’an station. A plot of spring phenological attributes at the tips of the phylogenetic tree of 77 species in Xi’an. FLD; first leaf date, FFD; first flower date, FLD-S_T_; first leaf date temperature sensitivity, FFD-S_T_; first flower date temperature sensitivity, FLD-S_Prc_; first leaf date precipitation sensitivity, FFD-S_Prc_; first flower date precipitation sensitivity, respectively. The sizes of the circles are proportional to the values of the characteristics, i.e., a larger size indicates later FLD or FFD, stronger temperature sensitivity (S_T_ with higher absolute value), and FLD-S_Prc_ more precipitation requirement. Bootstrap values of Neighbor Joining, Maximum Likelihood and Bayesian Interference are written above each branch respectively. Molecular divergence time and 95% HPD values are written below each branch.

Our molecular phylogenetic tree results demonstrate that closely related genera and species are located nearby in the topology (**Figures 4**, **5**). However, the sizes of the circles in front of each species reveal the traits’ values, i.e., a more significant size denotes later FLD or FFD, strong temperature sensitivity, or a greater need for precipitation. We addressed the outcomes of substantial phylogenetic conservation in Xi’an species here. Our findings show that the values of FFD, FFD-S_T_, and FFD-S_Prc_ of Xi’an species are more robust (**Figure 5**). High K values illustrate phylogenetic conservatism in FFD, FFD-S_T_, and FFD-S_Prc_ in Xi’an. However, phylogenetic signals in the timing of spring phenology were not significantly different from these findings. These findings indicated that the phylogenetic conservatism is more significant and stable in the Xi’an species FFD, FFD-S_T_, and FFD-S_Prc_ (**Table 1**; **Figure 5**). In contrast, the findings of the Guiyang species are identical, except that FLD-S_Prc_ results demonstrate stronger precipitation sensitivity (**Table 1**; **Figure 4**). According to different site ecologies, species sample sizes, and the accuracy of the underlying site-level phylogenetic trees, significance differed between sites. This variation is most likely due to these factors.

### 3.4 The evolutionary history of Xi’an and Guiyang species

With strong bootstrap support values, both phylogenetic trees developed larger bi-phyletic clades. We calculated the divergence periods for the biphyletic tree lineages in Guiyang to be 36 mya based on the cpDNA data. The molecular dating results suggested that the species in each clade appeared to have separated from their relatives during the middle Eocene in the Tertiary Cenozoic Era at 46.0 mya (**Figure 4**). According to the cpDNA findings, the divergence periods for Xi’an’s species’ significant biphyletic tree lineages were 76 mya. The molecular dating findings also indicated that the Xi’an species in each clade separated from its relatives during the late Cretaceous Mesozoic epoch (**Figure 5**).

### 3.5 Evolutionary patterns of phenological traits

**Figure 6:**
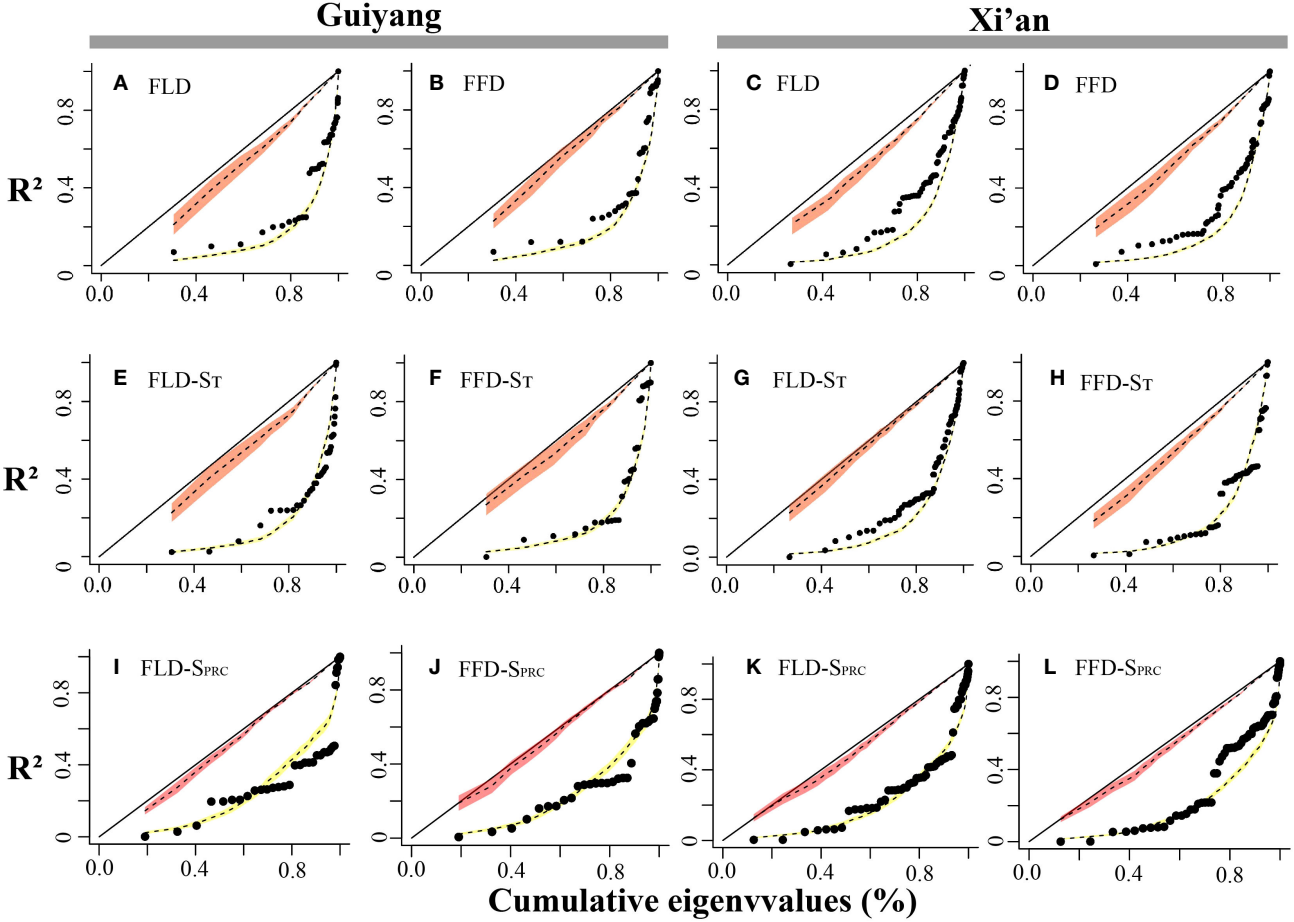
Spring phenological traits’ phylogenetic signal representation (PSR) curves. The red and yellow bands show the confidence intervals for the BM model and WN random expectations. We can find the 1:1 line in black. The black dots represent the phylogenetic eigenvectors that were consecutively added. The x-axes reflect the cumulative total of the eigenvalues, while the y-axes represent the R^2^ values of the successive PVR models. **(A)** FLD; first leaf date, **(B)** FFD; first flower date, **(C)** FLD; first leaf date, **(D)** FFD; first flower date, **(E)** FLD-S_T_; first leaf date temperature sensitivity, **(F)** FFD-S_T_; first flower date temperature sensitivity, **(G)** FLD-S_T_; first leaf date temperature sensitivity, **(H)** FFD-S_T_; first flower date temperature sensitivity, **(I)** FLD-S_Prc_; first leaf date precipitation sensitivity, **(J)** FFD-S_Prc_; first flower date precipitation sensitivity, **(K)** FLD-S_Prc_; first leaf date precipitation sensitivity, **(L)** FFD-S_Prc_; first flower date precipitation sensitivity, respectively.

The spring phenological trait evolution rate was not as steady as predicted by the Brownian model. PSR curves were higher than the null model but lower than the 45-degree reference line for all phenological characteristics. The findings indicated that the black line in Guiyang was closer to the yellow line in each stage than in Xi’an, indicating that the development of spring phenological traits in Guiyang was more consistent with random shift and that phylogeny had a less significant impact than in Xi’an (**Figure 6**). Additionally, the pace of evolution is changing quickly. Although evolution progresses slowly at first, it accelerates quickly toward the conclusion of the curve. This demonstrated that younger species are evolving more rapidly in Xi’an and Guiyang than elder species.

### 3.6 Plant functional trait relationship with spring phenology

We investigated the functional characteristics (trees and shrubs; biotic and abiotic pollination; deciduous and evergreen) that influenced the spring phenology of plants in Guiyang and Xi’an. Our findings indicated that the dominant tree species in Xi’an exhibit later leafing and early flowering. In Guiyang, tree species had leafed out (15.55 days) later than shrubs with a significant correlation, while the tree’s flowers bloomed (45 days) earlier than shrubs (**Table 2**). Trees predominate in Guiyang, suggesting leaf out and flowering would occur later. Similarly, the tree species of Xi’an also exhibit the same pattern, leaf out emerging (9.2 days) later and significantly correlated with shrubs. In comparison, flowering occurs (0.988 days) sooner in the trees than in shrubs in Xi’an (**Table 2**). The temperature and precipitation sensitivity of leaf out and flowering (FLD-S_T,_ FFD-S_T,_ FLD-S_Prc,_ and FFD-S_Prc_) in Guiyang species indicate non-significant results. While temperature sensitivity results of leaf out (FLD-S_T_) in Xi’an species show significant correlations, with a rise of 1°C, trees’ reaction to temperature is 0.657 days/°C later than shrubs. On the other hand, the temperature sensitivity of flowering (FFD-S_T_) and precipitation sensitivity of leaf out and flowering (FLD-S_Prc_ and FFD-S_Prc_) in Xi’an species reveal a non-significant correlation.

**Table 2:** The relationship between plant spring phenophases and sensitivity of spring phenophases with plant functional trait based on phylogenetic generalized least squares (PGLS) models.

Area	Functional Traits	Phenological Traits	Coefficient	P Value
Xi’an	Life Form (Trees and Shrubs)	FLD	9.2	<0.05*
		FFD	-0.988	0.925
		FLD-S_T_	0.657	0.002*
		FFD-S_T_	-0.367	0.602
		FLD-S_Prc_	0.029	0.1739
		FFD-S_Prc_	0.014	0.549
Guiyang	Life Form (Trees and Shrubs)	FLD	15.5476	<0.01*
		FFD	-45	0.214
		FLD-S_T_	0.322	0.688
		FFD-S_T_	-1.2833	0.6125
		FLD-S_Prc_	-0.001	0.98
		FFD-S_Prc_	0.015	0.763
Xi’an	Pollination Form (Biotic Vs Abiotic)	FLD	3.786	0.179
		FFD	-17.319	0.09
		FLD-S_T_	0.178	0.417
		FFD-S_T_	-0.402	0.5608
		FLD-S_Prc_	0.0274	0.156
		FFD-S_Prc_	-0.084	0.001
Guiyang	Pollination Form (Biotic Vs Abiotic)	FLD	7.19	0.169
		FFD	-22.821	0.453
		FLD-S_T_	0.473	0.477
		FFD-S_T_	-0.706	0.738
		FLD-S_Prc_	0.030	0.187
		FFD-S_Prc_	0.054	0.034
Xi’an	Species Group (Evergreen Vs Deciduous)	FLD	2.046	0.214
		FFD	57.721	<0.02*
		FLD-S_T_	-0.53	0.688
		FFD-S_T_	4.626	<0.01*
		FLD-S_Prc_	-0.009	0.98
		FFD-S_Prc_	-0.016	0.763
Guiyang	Species Group (Evergreen Vs Deciduous)	FLD	-5.31	0.321
		FFD	48.48	0.925
		FLD-S_T_	-0.006	0.652
		FFD-S_T_	0.304	0.602
		FLD-S_Prc_	0.038	0.1739
		FFD-S_Prc_	-0.046	0.549

FLD, first leaf date; FFD, first flower date; FLD-S_T_, first leaf date temperature sensitivity; FFD-S_T_, first flower date temperature sensitivity; FLD-S_Prc_, first leaf date precipitation sensitivity; FFD-S_Prc_, first flower date precipitation sensitivity. * Means significant value.

Regarding pollination types (biotic and abiotic pollination of plants), we discovered that flowering phenology (FFD) and temperature-sensitive flower phenology (FFD-ST) interacted with a non-significant but strong relationship with biotic and abiotic pollination in Xi’an and Guiyang. At the same time, leaf-out phenology (FLD) and temperature-sensitive leaf phenology (FLD-ST) show a non-significant and weak relationship with abiotic and biotic pollination in Xi’an and Guiyang species. The wind strongly correlated with flowering phenology under climatic sensitivities (FFD-S_T_ and FFD-S_Prc_) (**Table 2**). These findings indicated that various pollination methods offer distinct correlation patterns with spring phenological features for the Guiyang and Xi’an species. Plants that receive both biotic and abiotic pollination produce leaves later but flowers earlier, with non-significant correlations (**Table 2**). The sensitivity of temperature and precipitation to leaf phenology reveals a weak relationship with biotic and abiotic pollination.

Our analysis of the distinct groups of plant species revealed a significant interaction between the flowering phenology in Xi’an and the mechanisms governing the species of evergreen and deciduous plants. Findings suggested that Guiyang species exhibit non-significant and weak correlations in all phenological traits with low values of the coefficient of PGLS. Contrarily, plant species in Xi’an show a different pattern with earlier leaf out in evergreen and deciduous plant species (2.046 days) and earlier flowering with significant and robust responses to evergreen and deciduous plant species (57.721* days) (**Table 2**). The temperature sensitivity findings of flowering (FFD-S_T_) in Xi’an species are (4.626* days/°C) stronger and significant reaction (**Table 2**). The results of the precipitation sensitivity test for leaf out and flowering in Guiyang and Xi’an species indicate a non-significant and stronger response with evergreen and deciduous plant species (**Table 2**).

**Figure 7:**
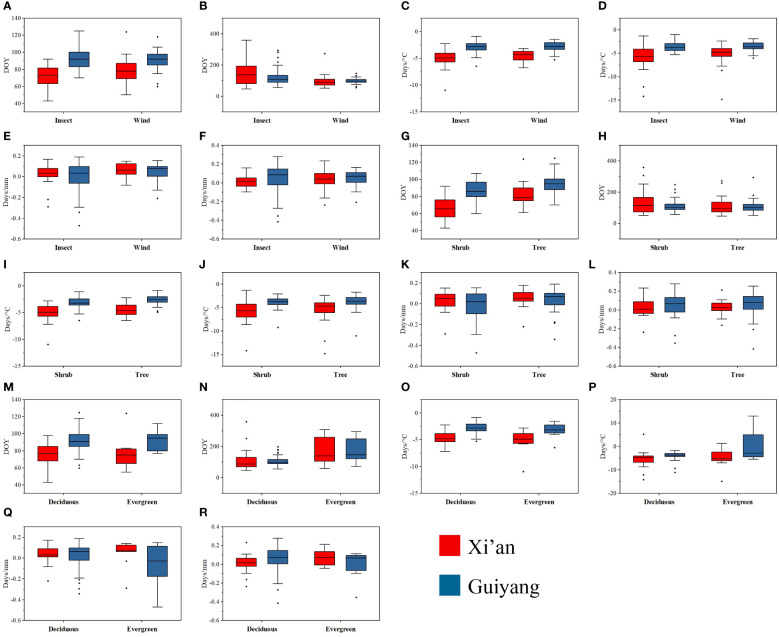
Mean and median range of spring phenological traits of tree and shrub species, biotic and abiotic pollination, and deciduous and evergreen species in response to first leaf dates, first flowering dates, temperature and precipitation sensitivity in Guiyang and Xi’an. **(A)** FLD of biotic and abiotic pollination species **(B)** FFD of biotic and abiotic pollination species **(C)** FLD-S_T_ of biotic and abiotic pollination species **(D)** FFD-S_T_ of biotic and abiotic pollination species **(E)** FLD-S_Prc_ of biotic and abiotic pollination species **(F)** FFD-S_Prc_ of biotic and abiotic pollination species **(G)** the FLD of tree and shrubs **(H)** FFD of tree and shrubs **(I)** FLD-S_T_ of tree and shrubs **(J)** FFD-S_T_ of tree and shrubs **(K)** FLD-S_PRC_ of tree and shrubs **(L)** FFD-S_PRC_ of tree and shrubs **(M)** FLD of evergreen and deciduous species **(N)** FFD of evergreen and deciduous species **(O)** FLD-S_T_ of evergreen and deciduous species **(P)** FFD-S_T_ of evergreen and deciduous species **(Q)** FLD-S_Prc_ of evergreen and deciduous species **(R)** FFD-S_Prc_ of evergreen and deciduous species. DOY: Day of the year, FLD; first leaf date, FFD; first flower date, FLD-S_T_; first leaf date temperature sensitivity, FFD-S_T_; first flower date temperature sensitivity, FLD-S_Prc_; first leaf date precipitation sensitivity, FFD-S_Prc_; first flower date precipitation sensitivity, respectively.

The mean and median range analysis revealed that similar phenological traits show a similar pattern in each functional attribute in both study areas, e.g., first leaf out dates (FLD) show a similar trend of mean and median range in each functional trait of Xi’an and Guiyang (**Figure 7**). The leaf out and flowering dates were counted as Days of Year (DOY), and the temperature sensitivity (days/°C) and precipitation sensitivity were shown as (days/mm) in **Figure 6**. The range of leaf-out phenology offers more than other phenophases in all functional traits, revealing that leaves take more time to bloom. Flowering phenology shows the minor range of mean and median in all available features except evergreen species. This represents more range, indicating that flowers take more time to bloom in the evergreen species group. The results also revealed that the mean and median of temperature sensitivity in all functional traits show the same pattern, indicating that temperature has equal importance in leaf and flowering phenology in all plant’s functional characteristics. On the other side, the mean and median of the precipitation sensitivity range show a different pattern in all functional features, representing that precipitation has different effects on each phenophases in each functional trait.

## 4 Discussion

### 4.1 Phylogenetic conservatism in plant phenophases

There was an association between phylogeny and the strength of the phenological shifts (Cohen et al., 2018; Inouye, 2022). The phylogenetic conservation in plant phenophases varies in different geographical and climatic conditions. In this study, we examined the correlations between plant spring phenology and the phylogeny of plant species in response to climatic and functional characteristics in Xi’an and Guiyang, China. Results revealed that phylogenetic signals in spring phenological traits were significantly conserved in Xi’an but non-conservative in Guiyang (**Table 1**). Significant phylogenetic signs in the Xi’an plant’s phenological characteristics indicate that closely related species typically have similar climatic adaptability for phenology (Davies et al., 2013). However, our findings demonstrated that the spring phenological features (FLD and FFD) showed non-conservative phylogenetic signals in Guiyang (**Table 1**), which was comparable with the results of some studies across the Tibetan Plateau, demonstrating the absence of phylogenetic signals in leaf unfolding (Yang et al., 2021). Our finding also indicated that the Guiyang species’ phylogenetic signs of blooming features were considerably more significant than those of leaf-out traits. Contrarily, leaf unfolding, a photosynthetic characteristic, may maximize environmental resources for promoting reproductive development (Gougherty and Gougherty, 2018). Consequently, it may be more responsive to environmental changes than blooming characteristics. These correlations between structural and functional variables during evolution may have stabilized environmental circumstances and shaped flowering traits (Memmott et al., 2007).

### 4.2 Phylogenetic conservation in climatic sensitive spring phenological traits

An emerging result of phylogeography and plastic responses to environmental cues specific to a particular area, like temperature, precipitation, and photoperiod, were phylogenetic signals. Each site exposes its species to the same set of environmental stimuli. We observed considerable variation in strength of conservatism between temperature and precipitation-sensitive spring phenophases (FLD-ST, FFD-ST, FLD-SPrc, and FFD-SPrc), possibly reflecting variation in climatic differentiation. Significant phylogenetic signals were found in Xi’an species in temperature and precipitation-sensitive spring phenology (FLD-ST, FFD-ST, FLD-SPrc, and FFD-SPrc), indicating that species were consistent with the climate change factors. Nonetheless, in certain instances, the degree of phylogenetic conservatism for mean FLD and FFD was higher within sites than seen worldwide. Phylogenetic conservatism was frequently weaker within an area than across locations. Our findings point to a significant intrinsic evolutionary conservatism in the Xi’an species’ phenological features, particularly noticeable when the species are subjected to similar external environmental stimuli. Recently, another study also reported the evolutionary signal of the temperature response of flowering time in Northeast China (Du et al., 2017). However, our findings demonstrated that the climatic sensitive spring phenological features (FLD-S_T_, FFD-S_T_, FLD-S_Prc_, and FFD-S_Prc_) showed non-conservative phylogenetic signals in Guiyang, which was similar to the results of spring phenological elements (FLD and FFD) (**Table 1**). Some studies in the Colorado Rocky Mountains (Caradonna and Inouye, 2015) demonstrated the absence of a phylogenetic signal (i.e., non-conservative phylogenetic signal) in the temperature sensitivity of spring phenological time. In comparison, the fact that the Rocky Mountains in Colorado have a harsher environment as the area under study and the solid abiotic selection pressures may restrict species growth and reproduction (Cavender-Bares et al., 2009; Lessard-Therrien et al., 2014). Their evolution is restricted in response to temperature and insignificant signals in temperature sensitivity species (Du et al., 2015; Basnett et al., 2019). The temperature and precipitation susceptibility of spring phenology in Guiyang species also showed no evidence supporting a phylogenetic signal, the same as spring phenology results. These findings demonstrated that the environmental (temperature and precipitation) and geographical circumstances (altitude) of the two regions (Xi’an and Guiyang) are distinct from one another and contribute to the variation in phylogenetic conservation in the spring phenology of plants.

Our findings suggest significant phylogenetic evidence in temperature and precipitation sensitivity phenological traits of Xi’an species while non-conservative in Guiyang plant species (**Table 1**), indicating species consistent with the studies for subtropical research (Li et al., 2020). Results revealed that the temperature of both areas is the same, but the precipitation difference is double that of Guiyang and Xi’an. Körner and Basler (Körner and Basler, 2010) previously reported that in most temperate tree species, phenological events such as flowering and autumnal cessation of growth are not primarily controlled by temperature. It was also suggested that phenology might be less sensitive to temperature and photoperiod and more tuned to seasonal shifts in precipitation (Reich, 1995; Morellato, 2003; Sanchez-Azofeifa et al., 2013). Such modifications are expected to occur in concert with rising global temperatures, but the direction and magnitude of change vary regionally (Cubasch et al., 2001). A recent study demonstrated that early flowering advanced under warming plus precipitation addition compared to warm, dry, mild, and very wet species (Ganjurjav et al., 2020). The advancement of spring phenology due to temperature and precipitation leads to evolutionary progress in phenological parameters that disturb the phylogenetic conservation of Guiyang species. The observations of weak conservatism have at least three plausible explanations. First, there is a chance that inaccurate evolutionary reconstructions or measured attributes will muddle the potential signal. Second, phenological cycles could evolve in a way that is poorly anticipated by phylogeny, for instance, when local adaptation or a comparable directional selection force is powerful and controls evolutionary trajectories. Third, phenology may show a flexible response to the environment independent of taxonomic membership (i.e., phenological plasticity), such that the environmental conditions largely determine the phenological schedules for species and populations.

### 4.3 Climatic conditions impacted phylogenetic conservatism in plant phenophases

Our molecular dating results revealed that Xi’an plant species diverged from their ancestors during the late Cretaceous period (95 Mya), while the Guiyang species diverged during the middle Eocene Era (46 Mya) (**Figures 4**, **5**). During the Cretaceous, the study area experienced large-scale magmatic intrusion under the influence of the late Yunshan movement, which was characterized by a significant global greenhouse climate, with a significant decrease in temperature and an increase in sea level (Wang et al., 2022). Previously it was also found that the Eocene–Oligocene transition in south-eastern Tibet changed the climate from sub-tropical/warm temperate to cool temperate, likely reflective of both uplift and secular climate (Su et al., 2019). We proposed that the recent elevation of the Tibet Plateau influences the climatic shift that led to the phylogenetic divergence of Guiyang species from their parents and to non-conservative phylogenetic signals. In contrast, Xi’an species have a long evolutionary history, making them more climate-adaptive and exhibiting notable phylogenetic conservation.

However, our findings revealed significant phylogenetic signals in spring phenology (FLD and FFD) and climate sensitivity (FLD-S_T_, FFD-S_T_, FLD-S_Prc_, and FFD-S_Prc_) in Xi’an species, while non-conservative in Guiyang. The long evolutionary history of Xi’an species suggested that plants are comparatively more stable and conserved than Guiyang species. For example, it has long been assumed, and sometimes demonstrated, that within a habitat type, the amount of ecological differentiation among species is proportional to the amount of evolutionary and genetic divergence (Stephens and Wiens, 2004). Ecological differentiation can result in reduced resource use overlap between species, allowing species to stabilize in new habitats slowly. Hence, the phylogenetic conservation in spring phenological traits is significant and more substantial in Xi’an after ecological differentiation in new habitats. Guiyang species have recently diverged and show non-significant conservation in plant spring phenology.

Another reason for non-conservative Guiyang species is that they typically have harsh climatic and geographical conditions such as higher altitude, high precipitation, high relative humidity, long, cloudy, rainy days, and little sunshine (**Figure 2**). Previously, it was reported that harsher settings might cause features among lineages not closely related to converging, diminishing the phylogenetic signal (Lessard-Therrien et al., 2014; Du et al., 2015). In contrast, a report suggested that some tree species showed a significant relationship between phylogenetic conservatism and plant phenology at high altitudes under harsh climates (Li et al., 2016). These phylogenetic linkages may be the basis for species-specific phenological sensitivity to abiotic variation and may aid in predicting these responses to climate change (Caradonna and Inouye, 2015). We also assume that natural selection and their genetic link were strongly correlated with species spread.

The phylogenetic signals of spring phenology were significantly conserved in Xi’an, which was highly consistent with the findings of other studies in Europe and North America (Davies et al., 2013), and China (Du et al., 2015, 2017; Li et al., 2016). This finding further supports the phylogenetic constraint theory in temperature and precipitation sensitivity for flowering. A similar phenomenon is observed in northern Europe, where tree species richness is low during glaciations and postglacial dispersal limitations (Svenning and Skov, 2007). Finally, it is worth mentioning that the combined effect of both the contemporary environment and historical contingencies is significant (Hawkins et al., 2003; Montoya et al., 2007). This is mainly because environmental conditions vary across biogeographical regions. The different combinations of local climatic and geographical variables can result in remarkable changes in plant phenological features. These findings support our hypothesis that local environmental adaptation (LEA) changes with geographical variations directly related to regional climatic conditions and affect the relationship between plant spring phenology and phylogeny. Furthermore, considerable conservation regimes in these regions should consider species diversity and their unique ecological and evolutionary history (Forest et al., 2007). The effects of historical contingencies are partly complicated by the contemporary environment (especially climate). We believe that the impact of the modern environment on plant phenology is fundamental across the globe, dominating general trends and that historical contingency may only cause deviations in phylogenetic conservation in both regions of Xi’an and Guiyang.

### 4.4 Interaction between plant functional traits and spring phenophases

This work examined the spring phenology in Xi’an (temperate) and Guiyang (subtropical) for species concerning phylogeny and functional features. For instance, our study discovered a strong and significant correlation between spring phenology and plant functional traits, such as life forms and distinct groups of plants (deciduous and evergreen) species in Xi’an (**Table 2**). The time of leaf expansion is significant in shrubs and earlier than that of trees in Xi’an, which is consistent with the research conclusion of Panchen et al (Panchen et al., 2015). in 8 botanical gardens in temperate regions of the northern hemisphere. The probable cause is that earlier leaf expansion allows shrubs to take full advantage of light for photosynthesis before the tree canopy is fully formed (Rollinson and Kaye, 2012; Panchen et al., 2015). The study also found a significant relationship between trees and earlier flowering onset in Xi’an, similar to the research conclusion (Du et al., 2017). Our results also revealed a significant relationship between deciduous and evergreen plant species with Xi’an flowering phenology, as suggested in previous research (Alice Boyle and Bronstein, 2012; Wang et al., 2020). Our pollination analysis results are not significant (**Table 2**), which show two possible explanations: First, wind-pollinated tree species need to bloom before the canopy closes, thereby reducing the blocking of leaves on the wind-borne pollen (Fenner, 1998); Second, the Guiyang and Xi’an regions early spring is relatively dry, cold and windy, which restricts the activities of pollinators, resulting in rather a late flowering in plants (Griz and MaChado, 2001). Additionally, our findings showed that trees have an advantage over shrubs in light interception and wind pollination for enhancing reproductive success (Alice Boyle and Bronstein, 2012).

Climatic conditions considerably impacted species’ flowering phenology differences (Chang-Yang et al., 2013; Qi et al., 2015). Functional features in this situation might operate as a steppingstone variable in the interaction between climatic conditions and spring phenology. Trees are more sensitive to temperature change than shrubs because of long-term environmental selection (Chávez-Pesqueira and Núñez-Farfán, 2016; Yang et al., 2018). However, our results support the association between growth type and spring phenology in Xi’an temperate climate zones. Temperature-sensitive results with life forms (trees and shrubs) and distinct groups (deciduous and evergreen) of plant species show a significant relationship with leaf and flower phenology in Xi’an, respectively. The above research conclusions reflect plant species’ different functional trade-off strategies that respond to the external environment and reflect the differentiation of plant niches and species to a certain extent. This suggests that more specific traits better suited for one particular region be used in the future. We consider identifying the most adaptative characteristics for surviving plant species in different biomes a vital goal. Finally, this study also has certain uncertainties, such as being limited by observation records. These plants usually span multiple families, resulting in insufficient resolution of the phylogenetic tree, which affects the accuracy of research conclusions to a certain extent. In the future, it is necessary to strengthen the phenological observation of more species records in the Xi’an and Guiyang area and refine the analysis to a few families to explore the plants more deeply and accurately.

## 5 Conclusions

This study examines the phylogenetic conservation between plant phenological traits and their relationship with biological characteristics. It uses first leaf and flower date data from the China Phenological Observation Network (CPON) for 77 and 40 plant species from Xi’an and Guiyang, respectively. We have shown that the initial leaf unfolding and blooming dates of plants in X’ian exhibit significant phylogenetic signals and are compatible with the OU evolutionary process; however, plant species show non-significant phylogenetic conservation in Guiyang. Similar to this, Xi’an species were significantly phylogenetic conserved with temperature and precipitation sensitivity of phenological traits (FLD-ST, FFD-ST, FLD-SPrc, and FFD-SPrc) but non-conservative in Guiyang. Our results suggested a strong relationship between FFD and FFD-S_T_ with plant functional traits of distinct plant groups (evergreen and deciduous) and life forms (trees and shrubs). These findings indicated that ecological and evolutionary processes under climate change and natural selection forces affect the phylogenetic conservation of the above phenological characteristics. Our results extended the basic phenology theory, providing a new perspective for correctly evaluating the relationship between climatic conditions and phylogenetic conservation with plant phenology.

## Data availability statement

The original contributions presented in the study are included in the article/**Supplementary Material**. Further inquiries can be directed to the corresponding authors.

## Author contributions

KS: Conceptualization, Formal analysis, Investigation, Methodology, Software, Writing – original draft, Writing – review & editing. MZ: Conceptualization, Data curation, Formal analysis, Software, Writing – review & editing. LC: Data curation, Formal analysis, Investigation, Software, Writing – review & editing. YH: Data curation, Investigation, Methodology, Software, Writing – review & editing. YZ: Data curation, Formal analysis, Methodology, Software, Writing – review & editing. WL: Data curation, Methodology, Software, Writing – review & editing. JD: Conceptualization, Funding acquisition, Project administration, Supervision, Writing – original draft, Writing – review & editing.
