# Supplementary material for: Phylogenetic conservation in plant phenological traits varies between temperate and subtropical climates in China
Source: Front Plant Sci. 2024 Apr 10;15:1367152. doi: 10.3389/fpls.2024.1367152 (PMC11039852; doi:10.3389/fpls.2024.1367152)
Supplement: Supplementary file 1 [file DataSheet_1.pdf]

Table S1. The detail of plant species, NCBI accession number, and functional traits (life forms, pollination style, and evergreen or deciduous species).

No.	Species Name	NCBI number	Type of plant	Pollination style	
**Guiyang**					
1	*Lagerstroemia indica*	KF572028	Trees	Insect	Deciduous
2	*Wisteria sinensis*	MN311170	Trees	Insects	Deciduous
3	*Cercis chinensis*	MZ128523	Shrubs	Insects	Deciduous
4	*Catalpa ovata*	MW493387	Trees	Insect	Deciduous
5	*Distylium dunnianum*	MW248109	Shrubs	Wind	Evergreen
6	*Ziziphus jujuba*	KX266829	Trees	Wind	Deciduous
7	*Magnolia denudata*	JX280394	Shrubs	Insects	Deciduous
8	*Ulmus pumila*	MW279236	Trees	Insects	Deciduous
9	*Camellia oleifera*	MF541730	Trees	Insects	Evergreen
10	*Zanthoxylum simulans*	MF716524	Shrubs	Insects	Deciduous
11	*Cedrus deodara*	NC_014575	Trees	Wind	Evergreen
12	*Ligustrum quihoui*	MN510462	Shrubs	Insects	Evergreen
13	*Populus adenopoda*	KX425622	Trees	Wind	Deciduous
14	*Cinnamomum camphora*	MH356726	Trees	Wind	Evergreen
15	*Lindera communis*	MT621585	Trees	Wind	Evergreen
16	*Malus micromalus*	MF062434	Shrubs	Wind	Deciduous
17	*Firmiana platanifolia*	MH671308	Trees	Insects	Deciduous
18	*Chaenomeles speciosa*	MT937182	Shrubs	Wind	Deciduous
19	*Amygdalus persica*	OK545754	Trees	Wind	Deciduous
20	*Pyrus pyrifolia*	NC_015996	Trees	Insects	Deciduous
21	*Cerasus yedoensis*	KU985054	Trees	Insects	Deciduous
22	*Celtis sinensis*	MN877379	Trees	Insects	Deciduous
23	*Vitis vinifera*	MN561034	Shrubs	Wind	Deciduous
24	*Eriobotrya japonica*	KT633951	Trees	Insects	Evergreen
25	*Ligustrum lucidum*	MH559273	Shrubs	Insects	Evergreen
26	*Osmanthus fragrans*	MH687871	Shrubs	Insects	Evergreen
27	*Hibiscus syriacus*	OM731671	Shrubs	Insects	Deciduous
28	*Chaenomeles sinensis*	MN506262	Shrubs	Wind	Deciduous
29	*Hibiscus mutabilis*	MK820657	Shrubs	Insects	Deciduous
30	*Chaenomeles cathayensis*	MN506260	Shrubs	Insects	Deciduous
31	*Pinus massoniana*	MF564195	Trees	Wind	Evergreen
32	*Quercus acutissima*	NC_039429	Trees	Wind	Deciduous
33	*Photinia davidsoniae*	MT230547	Trees	Insects	Evergreen
34	*Melia azedarach*	MT460410	Trees	Wind	Deciduous
35	*Prunus salicina*	MH700952	Trees	Insects	Deciduous
36	*Chimonanthus praecox*	MT859152	Shrubs	Insects	Deciduous
37	*Forsythia viridissima*	MW856917	Shrubs	Insects	Deciduous

**Xian**					
1	*Alangium chinense*	MG524996	Trees	Insects	Evergreen
2	*Syringa oblata var. alba*	ON920512	Shrubs	Insects	Deciduous
3	*Betula platyphylla*	MH205735	Trees	Wind	Deciduous
4	*Fraxinus chinensis*	MW599993	Trees	Wind	Deciduous
5	*Syringa pekinensis*	MN901632	Shrubs	Insects	Deciduous
6	*Ailanthus altissima*	MG799542	Trees	Insects	Deciduous
7	*Salix babylonica*	MF189167	Trees	Insects	Deciduous
8	*Robinia pseudoacacia*	MT120809	Trees	Insects	Deciduous
9	*Eucommia ulmoides*	MF766010	Trees	Wind	Deciduous
10	*Pterocarya stenoptera*	MN866892	Trees	Wind	Deciduous
11	*Broussonetia papyifera*	MF496038	Trees	Insect	Deciduous
12	*Malus spectabilis*	MT501657	Trees	Insects	Deciduous
13	*Clerodendrum trichotomum*	MT473746	Shrubs	Insects	Evergreen
14	*Albizia julibrissin*	MW539046	Trees	Insects	Deciduous
15	*Juglans regia*	KT870116	Trees	Wind	Deciduous
16	*Quercus aliena*	KU240007	Trees	Wind	Deciduous
17	*Zanthoxylum bungeanum*	MW602886	Shrubs	Wind	Deciduous
18	*Syringa persica*	MH817880	Shrubs	Insects	Deciduous
19	*Phellodendron amurense*	KY707335	Trees	Wind	Deciduous
20	*Rosa xanthina*	MT547539	Shrubs	Wind	Deciduous
21	*Pistacia chinensis*	MT157378	Trees	Wind	Deciduous
22	*Berberis amurensis*	KM057374	Shrubs	Insects	Deciduous
23	*Pyracantha fortuneana*	MW596361	Shrubs	Insects	Evergreen
24	*Acer palmatum var. palmatum*	MN864504	Trees	Insects	Deciduous
25	*Populus x canadensis*	MK267315	Trees	Wind	Deciduous
26	*Edgeworthia chrysantha*	MT135125	Shrubs	Insects	Deciduous
27	*Forsythia viridissima*	MW856917	Shrubs	Insects	Deciduous
28	*Weigela florida*	MG738664	Shrubs	insect	Deciduous
29	*Caragana sinica*	OL310480	Shrubs	Insects	Deciduous
30	*Diospyros lotus*	KM522849	Shrubs	Insects	Deciduous
31	*Forsythia suspensa*	MF579702	Shrubs	Wind	Deciduous
32	*Melia azedarach*	MT460410	Trees	Insects	Deciduous
33	*Koelreuteria paniculata*	KY859413	Trees	Insects	Deciduous
34	*Populus tomentosa*	MK252102	Trees	Wind	Deciduous
35	*Swida walteri*	MT816471	Shrubs	Insects	Deciduous
36	*Chaenomeles cathayensis*	MN506260	Shrubs	Insects	Deciduous
37	*Paeonia suffruticosa*	OK662586	Shrubs	Insects	Deciduous

38	*Chaenomeles sinensis*	MN506262	Shrubs	Wind	Deciduous
39	*Hibiscus syriacus*	OM731671	Shrubs	Insects	Deciduous
40	*Osmanthus fragrans*	MH687871	Shrubs	Insects	Evergreen
41	*Nandina domestica*	DQ923117	Shrubs	Wind	Evergreen
42	*Ligustrum lucidum*	MH559273	Shrubs	Insects	Evergreen
43	*Eriobotrya japonica*	KT633951	Trees	Insects	Evergreen
44	*Aesculus chinensis*	MK648235	Trees	Insects	Deciduous
45	*Morus alba*	MT577029	Shrubs	Wind	Deciduous
46	*Malus baccata*	MK571561	Trees	Insects	Deciduous
47	*Amygdalus davidiana*	MK798145	Trees	Insects	Deciduous
48	*Crataegus pinnatifida*	MN102356	Trees	Insects	Deciduous
49	*Cornus officinalis*	MH729079	Shrubs	Insects	Deciduous
50	*Photinia serrulata*	MZ153171	Trees	Insects	Evergreen
51	*Diospyros kaki*	MW557547	Trees	Wind	Deciduous
52	*Quercus variabilis*	MK105465	Trees	Wind	Deciduous
53	*Chaenomeles speciosa*	MT937182	Shrubs	Wind	Deciduous
54	*Cydonia oblonga*	MN061993	Shrubs	Insects	Deciduous
55	*Xanthoceras sorbifolia*	MN608158	Trees	Insects	Deciduous
56	*Sapindus mukorossi*	KM454982	Trees	Wind	Deciduous
57	*Firmiana platanifolia*	MN533966	Trees	Insects	Deciduous
58	*Acer pictum subsp. mono*	MK508999	Trees	Wind	Deciduous
59	*Toona sinensis*	OL693863	Trees	Insects	Deciduous
60	*Platanus occidentalis*	DQ923116	Trees	Wind	Deciduous
61	*Cedrus deodara*	NC_014575	Trees	Wind	Evergreen
62	*Ginkgo biloba*	MN443423	Trees	Wind	Deciduous
63	*Cerasus pseudocerasus*	KX255667	Shrubs	Insects	Evergreen
64	*Jasminum nudiflorum*	DQ673255	Shrubs	Wind	Deciduous
65	*Pinus tabuliformis*	KT740995	Trees	Insects	Evergreen
66	*Ulmus pumila*	MW279236	Trees	Insects	Deciduous
67	*Amygdalus triloba*	MK790138	Trees	Insects	Deciduous
68	*Magnolia denudata*	JX280394	Shrubs	Insects	Deciduous
69	*Ziziphus jujuba*	KX266829	Trees	Wind	Deciduous
70	*Kerria japonica c. pleniflora*	MN418902	Shrubs	Insects	Deciduous
71	*Amygdalus triloba f. multiplex*	MT937181	Trees	Insects	Deciduous
72	*Bischofia polycarpa*	MZ826267	Trees	Insects	Deciduous
73	*Syringa oblata*	MW464119	Shrubs	Insects	Deciduous
74	*Cercis chinensis*	MZ128523	Shrubs	Insects	Deciduous
75	*Amorpha fruticosa*	MN709789	Shrubs	Insects	Deciduous
76	*Wisteria sinensis*	MN311170	Trees	Insects	Deciduous
77	*Lagerstroemia indica*	KF572028	Trees	Insect	Deciduous

**Outgroups**				
1	*Agaricus bisporus*	AC253835.1	Fungi	Outgroup
2	*Cantharellus cibarius*	NC_020368.1	Fungi	Outgroup

INDEX I
Fossil Calibration

The approach (Marshall, 2008) involves generating an uncalibrated ultrametric tree concerning the fossil record and mapping all candidate fossil calibrations onto the tree to determine which of the calibrated lineages has the best temporal fossil coverage. Specifically, the method aims to identify the lineage for which the oldest fossil (for that lineage) sits proportionally closest to the node of its MRCA (true time of origin) and, therefore, has the best temporal coverage. In each case, the calibration set and the corresponding molecular dates from the single-fossil dating analyses were used by using the method of Near et al. (2005).

We conducted the cross-validations using both the mean and median age estimates to evaluate whether the posterior age distributions (rather than point age estimates) influenced which fossil calibrations were identified as incongruent. The difference between the molecular (MA) and fossil age (FA) at each node was calculated as Di = (Mai - FAi), where Fai is the fossil age, and Mai is the mean or median molecular age estimate for node i using the candidate fossil calibration at node x (see Near et al., 2005).

To stabilize estimated ages at deeper nodes, we constrained the root using a normal prior (mean = 110 MA, 95% confidence interval = 85-135 MA) spanning a wide range of plausible dates for all single-fossil calibration analyses (Vimoksalehi et al., 2012). BEAST analysis is used for molecular divergence analysis (See Material and Methods).

INDEX II
Phylogenetic tree reconstruction

Gene genealogies based on complete chloroplast genomes (cpDNA) were reconstructed by using MEGA v 7.0 and MrBayes v 3.2.3 (Ronquist & Huelsenbeck, 2003) to obtain the maximum likelihood (ML), maximum parsimony (MP), and Bayesian inference (BI) cladograms. To examine the phylogenetic relationships among the Guiyang and Xian plant species, we used the complete chloroplast genomes of all species (Supplementary Table S1), including outgroup taxa. jModelTest and PAUP* v 4.0 software were used

to obtain the general time-reversible model and gamma (G) distribution for the rate variation among sites based on Akaike's information criterion (Swofford, 2002). For the ML and MP phylogenetic cladogram analyses, we conducted 1,000 bootstrap replicates. To construct the best phylogenetic tree in Mr Bayes, we kept the burn-in at 2,500 and retained every 1,000 generations from 30,000,000 random tree rotations. The FigTree program was used to visualize the output (Rambaut, 2009).

References of Supplementary Information

1. Lukoschek, V., Scott Keogh, J. and Avise, J.C., 2012. Evaluating fossil calibrations for dating phylogenies in light of rates of molecular evolution: a comparison of three approaches. Systematic Biology, 61(1), p.22.
2. Marshall, C.R., 2008. A simple method for bracketing absolute divergence times on molecular phylogenies using multiple fossil calibration points. The American Naturalist, 171(6), pp.726-742.
3. Near, T.J., Meylan, P.A. and Shaffer, H.B., 2005. Assessing concordance of fossil calibration points in molecular clock studies: an example using turtles. The American Naturalist, 165(2), pp.137-146.
4. Rambaut, A., 2009. FigTree v1. 3.1 (http://tree. bio. ed. ac. uk/software/figtree/). Oxford.
5. Ronquist, F. and Huelsenbeck, J.P., 2003. MrBayes 3: Bayesian phylogenetic inference under mixed models. Bioinformatics, 19(12), pp.1572-1574.
6. Swofford, D. (2002). PAUP* Phylogenetic analysis using parsimony (* and other methods). Version 4. : Sinauer Associates.
